# Acute effects of high-intensity exercise on brain mechanical properties and cognitive function

**DOI:** 10.1007/s11682-024-00873-y

**Published:** 2024-03-28

**Authors:** Grace McIlvain, Emily M. Magoon, Rebecca G. Clements, Alexis Merritt, Lucy V. Hiscox, Hillary Schwarb, Curtis L. Johnson

**Affiliations:** 1 Department of Biomedical Engineering, University of Delaware, Newark, DE 19716, USA; 2 Department of Biomedical Engineering, Columbia University, New York, NY, USA; 3 Cardiff University Brain Research Imaging Centre (CUBRIC), Cardiff University, Cardiff, UK; 4 Department of Psychology, University of Nebraska-Lincoln, Lincoln, NE, USA

**Keywords:** Exercise, Elastography, Viscoelasticity, Executive Control, Cognition

## Abstract

Previous studies have shown that engagement in even a single session of exercise can improve cognitive performance in the short term. However, the underlying physiological mechanisms contributing to this effect are still being studied. Recently, with improvements to advanced quantitative neuroimaging techniques, brain tissue mechanical properties can be sensitively and noninvasively measured with magnetic resonance elastography (MRE) and regional brain mechanical properties have been shown to reflect individual cognitive performance. Here we assess brain mechanical properties before and immediately after engagement in a high-intensity interval training (HIIT) regimen, as well as one-hour post-exercise. We find that immediately after exercise, subjects in the HIIT group had an average global brain stiffness decrease of 4.2% (*p* < 0.001), and an average brain damping ratio increase of 3.1% (*p* = 0.002). In contrast, control participants who did not engage in exercise showed no significant change over time in either stiffness or damping ratio. Changes in brain mechanical properties with exercise appeared to be regionally dependent, with the hippocampus decreasing in stiffness by 10.4%. We also found that one-hour after exercise, brain mechanical properties returned to initial baseline values. The magnitude of changes to brain mechanical properties also correlated with improvements in reaction time on executive control tasks (Eriksen Flanker and Stroop) with exercise. Understanding the neural changes that arise in response to exercise may inform potential mechanisms behind improvements to cognitive performance with acute exercise.

## Introduction

Regular engagement in physical activity has profound effects on brain structure and function (Baek, 2016). Exercise protects tissue structural integrity against the negative effects of aging and neurodegenerative disease, including by preventing the loss of neurons, the decline of glial matrix integrity, and the loss of white matter fiber integrity (Dishman et al., 2006). These positive changes to brain tissue microstructure from exercise manifest as improvements to cognitive performance (Hillman et al., 2008). Exercise training therefore has the potential to be used as an accessible tool to counter the effects of neurodegeneration and functional impairment. Recently, it has been found that engagement in even a single session of exercise can boost cognitive performance and functional connectivity in the short term (Suwabe et al., 2018; Tomporowski, 2003; Voss et al., 2020). However, while these acute functional changes are increasingly evident, it remains unclear if and how brain structure is affected acutely by exercise. Using sensitive imaging metrics in vivo to measure exercise-induced changes in brain structure may enlighten the relationship between acute exercise and improvements to cognitive function, as well as informing intervention methods for preventing cognitive decline in older age.

One such imaging method is magnetic resonance elastography (MRE), which is an MRI technique to noninvasively measure tissue mechanical properties (Hiscox et al., 2016). MRE uses shear wave displacements to produce maps of brain mechanical properties, including stiffness and damping ratio; these measures can be calculated with high reliability even in small subregions of the brain (Delgorio et al., 2022; McIlvain et al., 2022c). Mechanical properties are uniquely sensitive to underlying tissue microstructure and it is hypothesized that mechanical properties relate to neuronal density, myelin integrity, composition of the glial matrix, and aspects of the cerebral vasculature (Guo et al., 2019; Hiscox et al., 2021; Murphy et al., 2019). MRE has revealed that changes in brain tissue mechanics occur during development (McIlvain et al., 2018) and aging (Sack et al., 2011), and that mechanical properties relate to individuals’ aerobic fitness (Schwarb et al., 2017) and body mass index (Hetzer et al., 2020). Recent evidence has shown that brain mechanical properties can positively change with intervention, as evidenced by a multi-week exercise trial in people with multiple sclerosis (Sandroff et al., 2017). Notably, MRE has been used to reveal that regional brain mechanical properties are sensitive to individual differences in cognitive function; for instance, the viscosity of the hippocampus is strongly related to episodic and relational memory performance (Hiscox et al., 2020; Schwarb et al., 2016). MRE even can outperform other imaging metrics in relating brain mechanical structure to individual cognitive function (Johnson et al., 2018). The sensitivity of mechanical properties to both cognitive function and aerobic fitness when measured cross-sectionally suggests that it may be a valuable tool for understanding brain structural dynamics during exercise.

During exercise, heart rate and vessel size increase, and there is decreased vascular resistance, changes to blood viscosity, and redistribution of relative blood flow (Moncion et al., 2022; Weng et al., 2016; X. Zhang et al., 2021). While MRE has not previously been used to measure changes during acute exercise, several studies have measured brain mechanical properties during other conditions of altered cerebral blood flow (CBF). The Valsalva maneuver (Herthum et al., 2021) and jugular compression (Hatt et al., 2015) are two mechanisms designed to increase intracranial pressure by increasing CBF and altering cerebrospinal fluid (CSF) dynamics. In a study of regional CBF and stiffness (Hetzer et al., 2018), increased intracranial perfusion was associated with an increase to cerebral stiffness. This finding was consistent with an animal study that showed increased intracranial pressure results in increases to brain stiffness (Arani et al., 2018). We expect that engagement in exercise will acutely alter brain mechanical properties (Ogoh & Ainslie, 2009), and we aim to understand if these mechanical changes are associated with the cognitive function improvements known to occur with acute exercise (Ai et al., 2021). Determining if brain mechanical properties can be acutely altered, and measuring if these changes relate to cognitive changes, will allow us to design interventions which target neuromechanical health.

## Methods

Thirty-six subjects (19 female, 17 male) between the ages of 19 and 30 years (mean = 22.7 ± 2.7 years), were recruited from the University of Delaware community. All subjects were cognitively normal, in good physical condition, and had no history of brain injury or central nervous system disease. The study population was restricted to young adulthood to avoid neurodegenerative pathology due to older age. The following racial groups were included in our study: Asian (*N* = 3), Black (*N* = 3), Mixed Race (*N* = 2), and White (*N* = 28); this distribution is consistent with the racial makeup of the University of Delaware community, The study protocol was approved by the University of Delaware Institutional Review Board and all subjects provided informed consent prior to participation.

Subjects were randomly divided into three groups, accounting for approximately even distribution of sex: two exercise groups and a control group. Six subjects were excluded from data analysis; one exercise subject failed to complete the full study protocol and five subjects (3 exercise, 2 control) were excluded for poor MRI image quality in one or more of their scans. The resulting sample included 30 subjects, with 10 subjects in the exercise group which completed the MRI scan first after exercise, 10 subjects in the exercise group which completed the behavioral assessments first after exercise, and 10 subjects in the control group that did not exercise. The control group was used to control for learning effects on the behavioral tasks. The different exercise groups were used to account for potential fading of exercise effects on brain or cognition while the other assessments were being performed.

**Fig. 1:**
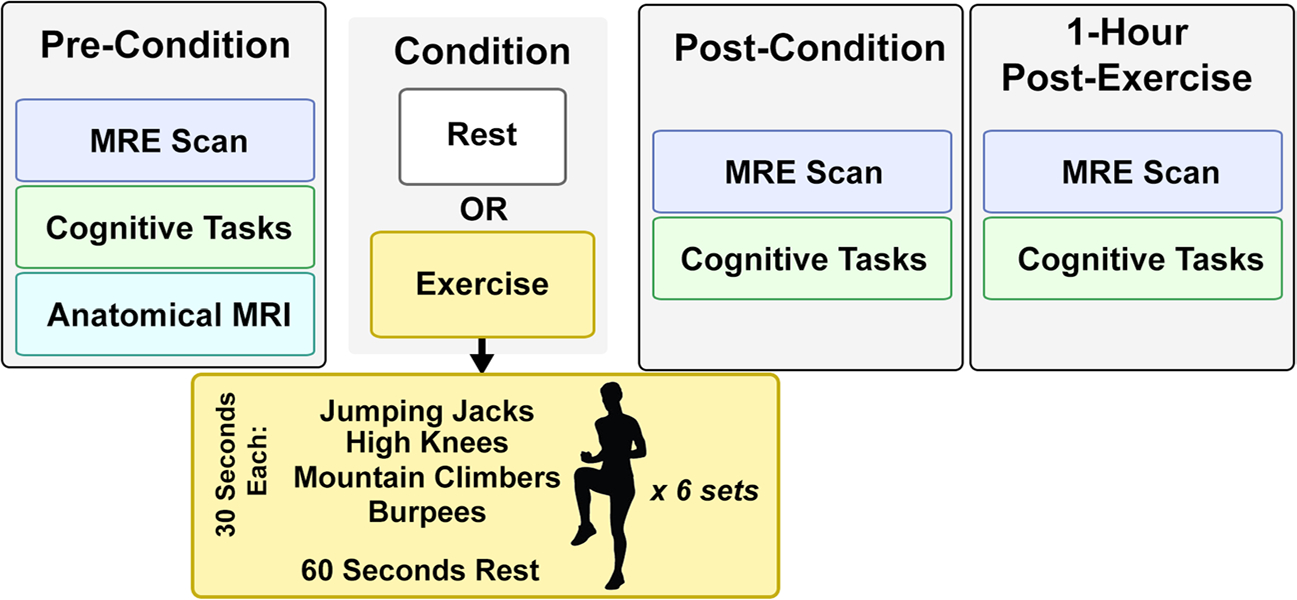
Overview of experimental procedure. Each subject was assigned to be part of an exercise group, which engaged in a high intensity interval training (HIIT) workout for 18 min, or a rest control group. All subjects had three sets of assessments which took place immediately before the condition, immediately after the condition, and one-hour post-condition, each of which included a high resolution MRE scan and cognitive assessments comprising the Stroop task and the Eriksen Flanker task. Additionally, an anatomical scan was collected at the first time-point to allow for regional segmentation during analysis

Figure 1 shows the data collection schedule, which consisted of three time points where MRI and cognitive data were collected. The first data point was a baseline assessment, which was followed by subjects completing the task for their assigned group: either exercise or control. Subjects in the control group sat in the waiting room for 18-min. Subjects in the exercise group completed an 18-min high-intensity interval training (HIIT) circuit. The HIIT circuit completed by the exercise group consisted of six sets of four 30-s exercises: jumping jacks, high knees, burpees, and mountain climbers, followed by a 60-s standing rest period in each set. Heart rate was measured at all data collection time points and periodically during exercise. Upon completion of exercise, all subjects in the exercise group had heart rates which exceeded 150 beats per minute, while the heart rates of subjects in the control group did not vary significantly from their baseline. The second data point was taken immediately after subjects completed their group condition, and the final data point was collected one-hour following the completion of the group condition. During the hour between the second and third data points, all subjects rested and did not engage in any exercise.

The order in which subjects completed the cognitive assessments or the MRI first after exercise was determined by their group and was varied between groups to account for the effects of exercise potentially fading with time. All subjects began their post-exercise assessment within 3 min of completing their final exercise set. Both the second MRI data set and the second cognitive test took no longer than 12 min each, and therefore all subjects entirely completed their second data collection within 25 min post-condition.

### Imaging protocol

MRE data was collected at each of the three time points on a Siemens 3 T Prisma MRI Scanner with a 64-channel head coil. The MRE scan was a 3D multishot, multiband, spiral sequence at 1.5 × 1.5 × 1.5 mm resolution with 50 Hz vibration delivered via pneumatic actuator with passive pillow driver (Resoundant, Inc; Rochester, MN). Data was undersampled using parallel imaging by a reduction factor R = 4, which involved undersampling two-fold in plane and two-fold through plane. Data was further undersampled spatiotemporally using a low-rank image and reconstruction approach, OSCILLATE (McIlvain et al., 2022a), to achieve an additional sampling reduction of R = 2. Additional imaging parameters included: field-of-view 240 × 240 mm^2^; matrix size 160 × 160; 80, 1.5 mm thick slices, TR/TE 2800/76 ms; bilateral, flow-compensated, matched-period motion-encoding gradients at 70 mT/m amplitude; and 4 evenly-spaced phase offsets. Each MRE scan took 4 min, 29 s. Each MRE scan also included an auxiliary sequence for coil sensitivity and magnetic field inhomogeneity mapping of matching matrix size, resolution, and slices with TR/TE1/TE2 of 4000/15/15.6 ms and took 1 min, 36 s (McIlvain et al., 2022b). An anatomical T1-weighted magnetization-prepared rapid acquisition gradient echo (MPRAGE) scan was also acquired during the first baseline imaging session with 0.9 × 0.9 × 0.9 mm^3^ resolution and TR/TI/TE = 2300/900/2.32 ms.

### Image processing

MRE imaging data was reconstructed in an iterative, joint, low-rank image reconstruction using a custom graphical processing unit (GPU) platform, PowerGrid (Cerjanic et al., 2016). Image reconstruction includes field inhomogeneity correction, SENSE parallel imaging, and correction for motion-induced phase errors between shots (Johnson et al., 2013). Data is reconstructed at a reduced rank of L = 12 from fully sampled rank of L = 24 using the OSCILLATE reconstruction scheme (McIlvain et al., 2022a). Phase unwrapping on reconstructed images was performed using PRELUDE in the FMRIB Software Library (FSL) (Jenkinson et al., 2012) and temporal Fourier filtering was used determine the complex amplitudes of the harmonic displacements.

A nonlinear inversion algorithm (NLI) (McGarry et al., 2012) was used to convert displacement fields into maps of viscoelastic shear stiffness and damping ratio. NLI calculates the complex viscoelastic shear modulus (G*=G’+iG”, where G’ is the storage modulus and G” is the loss modulus. Data is reported as composite measures of viscoelastic shear stiffness, μ, which is calculated as μ=2G*2/G’+G* (Manduca et al., 2001) and damping ratio, ξ, which is calculated as ξ=G”/2G’ (McGarry & Van Houten, 2008). These measures have been seen to reflect brain health and relate to cognitive function (Hiscox et al., 2020). For estimating properties of small anatomical regions, we used a soft prior regularization (SPR) (McGarry et al., 2013), which penalizes property variation over a priori regions during NLI optimization. SPR improves repeatability in small regions by reducing the variability that can arise from contamination by neighboring tissues and CSF. Datasets were also reviewed for motion artifacts, which we found occurred in some subjects at the second time point due to heavy breathing from exercise. Subjects who had motion artifacts at any time point were removed entirely from analysis (*N* = 5).

To create masks of regions-of-interest (ROIs) for analyzing MRE data, anatomical MPRAGE images were segmented in FreeSurfer v 6.0.0 (Fischl, 2012). Global ROIs included the whole cerebrum, cortical gray matter, white matter, and local ROIs included the hippocampus, thalamus, anterior cingulate cortex (ACC), and the dorsolateral prefrontal cortex (dlPFC). These regions were chosen due to known relationships with the cognitive tasks administered (Ando et al., 2020; Botvinick et al., 2004). Individual ROI masks were registered to MRE magnitude images using a linear affine transformation with 6-degrees of freedom in FSL FLIRT (FMRIB’s Linear Image Registration Tool) (Jenkinson et al., 2002). CSF was segmented from the MPRAGE using FAST (FMRIB’s Automated Segmentation Tool) (Zhang et al., 2001) in FSL. Voxels with greater than 1% CSF were excluded from regional masks, as CSF is not valid for mechanical property inversion. Regional mechanical properties were calculated by taking an average of the mechanical property maps over the area of the anatomical mask and averaged bilaterally.

### Behavioral task protocol

Two computerized behavioral tasks were used to assess cognitive function: the Eriksen Flanker task and the Stroop task. These tasks were completed in a separate room from the MRI scan. Both tasks measure reaction time in processing a target value among interfering stimuli. The Eriksen Flanker task presents subjects with a string of five characters: a centered stimulus with two flanking characters on either side. Trials are either congruent, where the stimulus matches the flanking characters (e.g. XXXXX), or incongruent, where the stimulus does not match the flanking characters (e.g. XXVXX). Subjects were asked to quickly respond with the centered stimulus by pressing the corresponding key on their keyboard. For the Stroop task, subjects were presented with the name of a color, and the color of the font either matched (congruent, e.g. RED, in red text) or did not match (incongruent, e.g. GREEN, in blue text) the word shown. Subjects were asked to quickly respond with the color of the text, not the name of the color written, by pressing the corresponding-colored key on their keyboard. Each session for both the Flanker and Stroop tasks consisted of 100 trials. Subjects practiced for both the Flanker and Stroop tasks with fifteen example trials before the first experimental trial. The Eriksen Flanker and Stroop outputs included reaction times of correct answers for both congruent and incongruent trials, and the Flanker or Stroop effect, which is the delay in reaction time that occur when processing incongruent stimuli as compared to congruent stimuli (i.e., difference in reaction time between congruent and incongruent trials). Reaction times for both the Flanker and Stroop tasks were z-scored relative to all subjects and time points were averaged together to create a composite reaction time. Behavioral data was assessed as Flanker effect, Stroop effect, and the average z-scored reaction times. All subjects participated in behavioral data collection at pre-condition and 1-h post-condition time points, but only *N* = 9 control subjects and *N* = 13 exercise subjects participated in behavioral data collection immediately after the condition (TP2).

### Statistics

Statistical analyses were conducted using Stata, version 17.1 (Statacorp, College Station, TX). Using a multivariate robust outlier approach to identify outliers (10% tail quantile), we found no MRE or cognitive data were outside three times the interquartile range and thus all data were included in the analysis. The association between the condition (exercise or rest) and “time” (pre-condition [TP1], immediately post-condition [TP2], and 1-h post-condition [TP3]) and the interaction between the two variables were assessed using a multilevel mixed-effects linear regression model adjusted for age and sex. Treatment group and time point were fixed effects, with participant ID modelled as a random effect to account for subject level variability. The restricted maximum likelihood method (REML) was used to produce unbiased estimates of variance and covariance parameters due to the sample size. If MRE measurements differed across time according to the intervention (i.e., a significant interaction), simple pairwise comparisons of contrasts were reported with Bonferroni correction for multiple ROIs. A similar approach was used to analyze the intervention and time effects for cognitive task performance. Separate models were run for stiffness and damping ratio and for each of the anatomical structures of interest (*n* = 7). Statistical significance for main and interaction effects were set at *p* < 0.05. Finally, change between the first and second time points was calculated for MRE measures and for z-scored reaction time, and a Pearson’s correlation was used across all subjects (exercise and control) to assess associations between mechanical property and cognitive function changes with exercise. A two-way analysis of variance (ANOVA) was used to determine if there were significant differences in measured brain mechanical properties depending on if subjects had the MRI scan or the cognitive assessments first after exercise; no significant differences were found (*p* = 0.341).

## Results

**Fig. 2:**
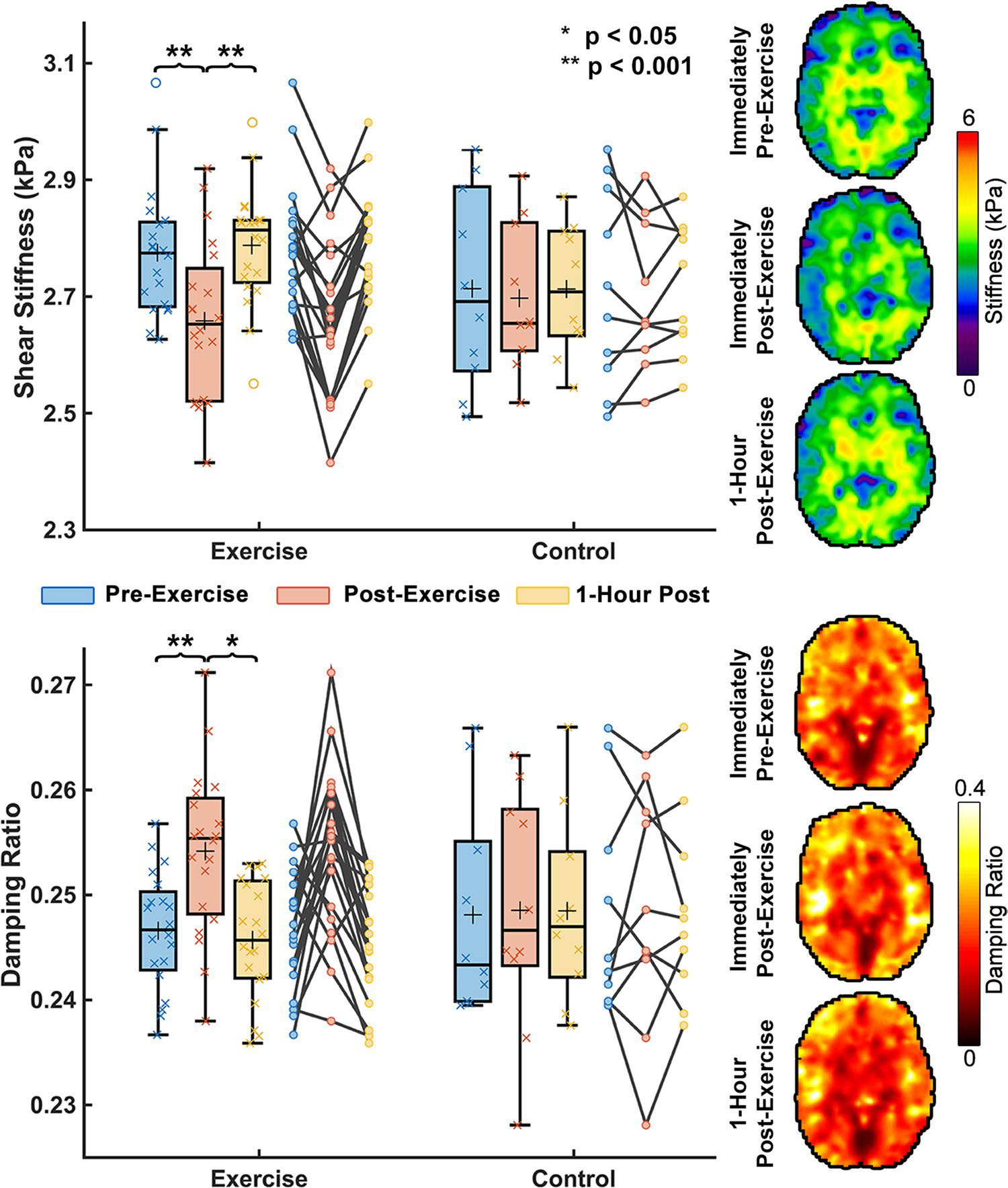
Whole brain mechanical property response to HIIT acute exercise. Brain mechanical properties were measured immediately pre-exercise, immediately post-exercise, and one-hour after completion of exercise. It was found that whole brain stiffness decreased by 4.2% immediately after exercise (*p* < 0.001) and whole brain damping ratio increased 3.1% (*p* = 0.002). One-hour post-exercise, both stiffness and damping ratio appeared to return to their baseline mechanical property values. Brain mechanical property maps are from a representative subject

A significant group x time point interaction was observed for both global brain stiffness (F = 13.91; *p* = 0.001) and damping ratio (F = 12.40; *p* = 0.002). Figure 2 shows an average brain stiffness decrease of 0.12 kPa or 4.2% (*p* < 0.001) between TP1 and TP2 in subjects who exercised. This stiffness decrease was found to be consistent across subjects with only 2 out of the 20 subjects not showing stiffness decreases; the maximum decrease in brain stiffness in any subject was 0.26 kPa or 9.8% from TP1 to TP2. Whole brain damping ratio increased on average 0.008 or 3.1% (*p* = 0.002). One-hour after exercise both stiffness and damping ratio returned to normal and were not significantly different than baseline values (TP1–3 μ: *p* = 0.508; ξ: *p* = 0.606). The control group showed no significant differences between any time points for stiffness (TP1: 2.71 kPa; TP2: 2.69 kPa; TP3: 2.71 kPa) or damping ratio (TP1: 0.248; TP2: 0.249; TP3: 0.249).

**Fig. 3:**
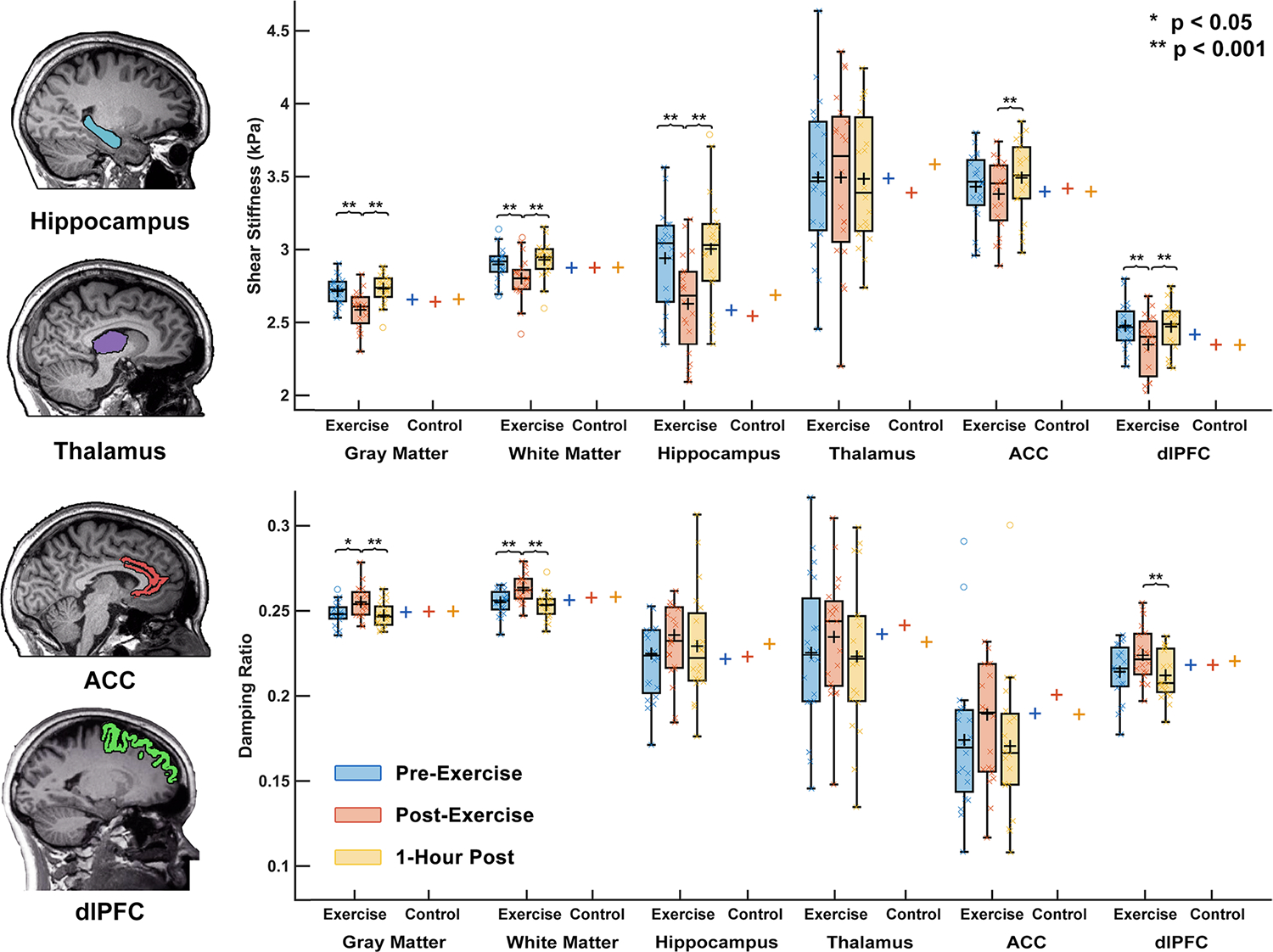
Regional brain mechanical property response to HIIT acute exercise including cortical gray matter, white matter, hippocampus, thalamus, anterior cingulate cortex (ACC), and dorsolateral prefrontal cortex (dlPFC). Brain mechanical properties were measured immediately pre-exercise, immediately post-exercise, and one-hour after completion of exercise. No significant changes with time point were found for the control group in any assessed brain region (box plots not shown)

Figure 3 shows mechanical property responses to exercise between brain regions. There was a significant group x time interaction observed in stiffness of cortical gray matter (F = 32.42, *p* < 0.001), white matter (F = 22.98, *p* < 0.001), hippocampus (F = 17.00, *p* < 0.001), ACC (F = 12.41, *p* = 0.002), and dlPFC (F = 28.37, *p* < 0.001). The hippocampus decreased in stiffness the most of any region with exercise, by 10.4%. In damping ratio, cortical gray matter (F = 10.27, *p* = 0.006) and white matter (F = 14.16, *p* = 0.002) changed significantly with exercise, but of the local regions only the dlPFC (F = 10.98, *p* = 0.004) changed significantly with exercise, increasing 5.1% between TP1 and TP2. The thalamus did not change significantly with exercise in either μ or ξ. One-hour after exercise the stiffness and the damping ratio of the cortical gray matter (μ: *p* = 0.210; ξ: *p* = 0.668), white matter (μ: *p* = 0.054; ξ: *p* = 0.343), hippocampus (μ: *p* = 0.474; ξ: *p* = 0.531), and dlPFC (μ: *p* = 0.727; ξ: *p* = 0.597) had returned to normal and were not significantly different than baseline.

**Fig. 4:**
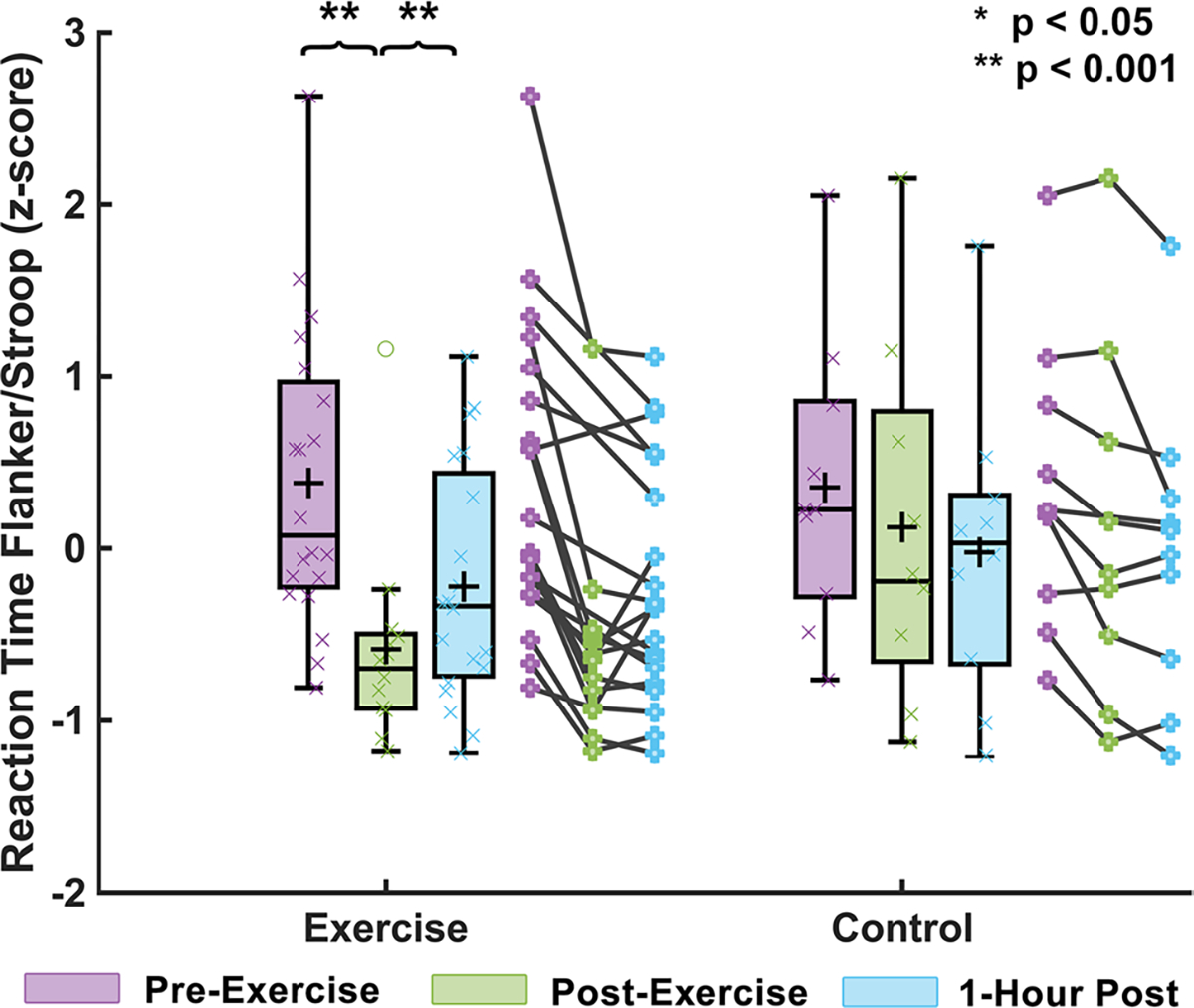
Cognitive function response to HIIT acute exercise. Cognitive tasks included the Flanker and Stroop tasks which measure executive control and reaction time by determining the ability and speed of a subject at identifying a target value among potential interfering stimuli

Figure 4 shows that combined reaction times from the Stroop and Flanker tasks differed between the exercise and control groups (F = 10.50, *p* = 0.005), indicating a significant influence of exercise on task performance. It is notable that the control group did improve in reaction time across points, likely due to training effects, however, the changes seen between TP1 and TP2 for the control group (Flanker RT: congruent 4.6%, incongruent 1.7%; Stroop RT: congruent 2.4%, incongruent 3.7%) were far less than changes in the exercise group (Flanker RT: congruent 11.6%, incongruent 10.9%; Stroop RT: congruent 15.3%, incongruent 14.2%). No significant differences were found for the group × time interaction in the Flanker effect (F = 1.30, *p* = 0.521) or Stroop effect (F = 1.45, *p* = 0.485) individually.

**Fig. 5:**
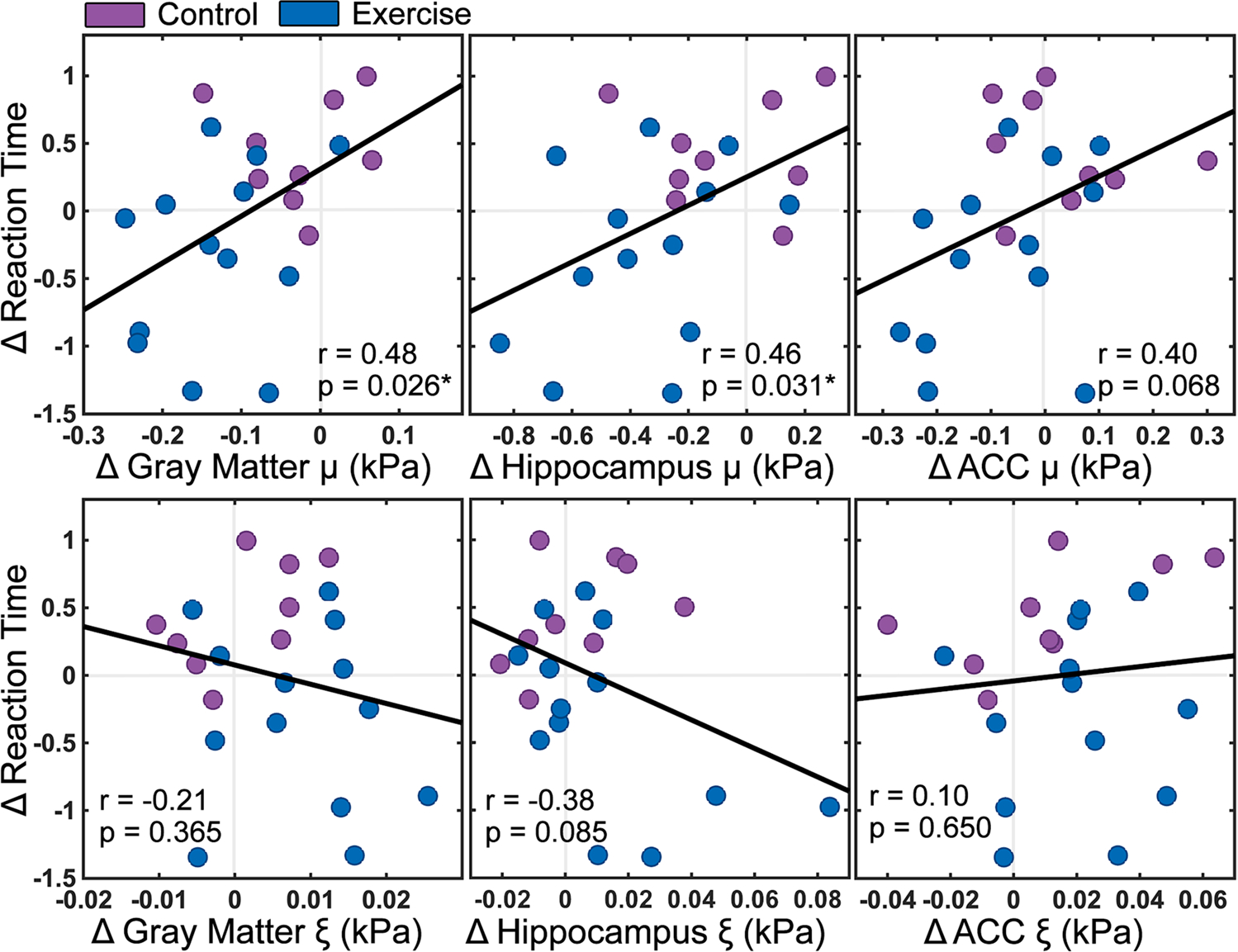
Relationships between changes to z-scored reaction time before and after exercise (TP1 to TP2) and changes (Δ) to mechanical properties including stiffness (*μ*) and damping ratio (*ξ*) in cortical gray matter, hippocampus, and anterior cingulate cortex (ACC)

Notably, Fig. 5 shows that changes to reaction time and changes to brain stiffness between TP1 and TP2 were significantly correlated with one another, such that greater decrease in stiffness correlated with a greater reduction in reaction time. Including all subjects, significant correlations were found between changes in reaction time and change in stiffness in the cortical gray matter (r = 0.48; *p* = 0.026) and the hippocampus (r = 0.46; *p* = 0.031), while the ACC was seen to approach significance (r = 0.40; *p* = 0.068). In just the exercise group, correlation coefficients were similar to the whole model for cortical gray matter (r = 0.32), hippocampus (r = 0.45), and ACC (r = 0.39). Changes to reaction time were not related to changes in damping ratio for any structure.

## Discussion

In this work we find that engagement in high intensity exercise induces measurable changes in brain mechanical properties in the short term, such that stiffness decreases and damping ratio increases, and that these changes return to baseline with rest. The directionality of these changes was opposite of our initial hypothesis. We also saw that the magnitude of change to mechanical properties from exercise is regionally dependent and appears to correspond with concomitant improvements in reaction time on assessments of executive control. Previous studies have demonstrated this acutely improved reaction time with exercise, but here we present evidence that this interaction may be influenced by changes to brain mechanical properties.

Only a handful of elastography studies have considered acute changes to brain mechanical properties. These include a study using hypercapnia to induce cerebral vasodilation and a study using the Valsalva maneuver to increase intracranial pressure (ICP), both of which observed an increase to brain stiffness of between 3 and 8% (Arani et al., 2018; Hatt et al., 2015; Herthum et al., 2021; Hetzer et al., 2018). Here we alter brain mechanical properties using high intensity exercise and observe acutely decreased global brain stiffness. High-intensity exercise is, to our knowledge, the first tested method for acutely *decreasing* brain stiffness. To date, only one study has considered the effects of exercise on brain mechanical properties and observed that brain stiffness *increased* over the span of a 12-week aerobic exercise intervention in people with multiple sclerosis (Sandroff et al., 2017); this chronic effect is the opposite of what we see during acute exercise. In fact, higher brain stiffness is widely thought to indicate greater tissue health in studies of aging, neurodegeneration, and cognitive function (Hiscox et al., 2021; Murphy et al., 2019). We expect that the physiological mechanisms controlling tissue mechanical properties during chronic and acute exercise are likely different, and the mechanisms which affect brain mechanics chronically, potentially including neurogenesis and remyelination, are not the same as those affecting brain mechanics acutely, which may be more affected by CBF and ICP.

One mechanistic hypothesis of acutely altered brain mechanics is changes to CBF during and immediately after exercise. Changes to CBF with exercise depend on the intensity of exercise and on time point at which CBF is measured (i.e. during or after exercise) (Brisswalter et al., 2002; Querido & Shell, 2007; K. J. Smith & Ainslie, 2017). During moderate intensity exercise, there is increased heart rate, vasodilation, and increased CBF (Billinger et al., 2017; Ogoh & Ainslie, 2009). However, it is thought that high intensity exercise produces a different CBF response. During exercise that uses greater than approximately 60% of maximal oxygen uptake, CBF reduces to a pre-exercise level, possibly due to a hyperventilation-induced cerebral vasoconstriction which is caused by a reduction to the partial pressure of arterial carbon dioxide (Joris et al., 2018; Ogoh & Ainslie, 2009). An elevated heart rate, with pre-exercise CBF, means there is less blood in the brain at any given moment, which may contribute to the decreases in brain stiffness immediately after exercise. Damping ratio, an expected measure of tissue organization, is increased with exercise, which could be a result of constricted blood vessel walls pulling more tightly on surrounding glial matrix, thus altering glial matrix organization. Cerebrovascular effects of between 10% (Steventon et al., 2020) and 40% (Alfini et al., 2016; Smith et al., 2010) have been reported with exercise; given the percentage of intracranial volume that is vasculature, the 4.2% stiffness and 3.1% damping ratio changes with exercise we report here, make it plausible that mechanical property response is due either directly or indirectly to cerebrovascular changes. Previous work has shown intracranial vasculature returns to baseline between 30 and 40 min after exercise (MacIntosh et al., 2014; Steventon et al., 2020); likewise we see brain mechanical properties at their baseline values at the 1 h follow-up scan. Arterial-spin-labeling (ASL) would allow us to measure the effects of blood flow on mechanical properties with exercise, but due to the limited useable time window after intervention, ASL data was not collected as part of this study. Finally, with exercise there is a release of brain-derived neurotrophic factor and insulin-like growth factor-1, which are known to contribute to improved cognitive function (Dinoff et al., 2017; Ferris et al., 2007; Winter et al., 2007). The role of these neurotrophic factors on brain mechanical properties in both the short- and long-term are unknown and warrant further investigation.

A previous study on rats found that brain temperature increased with exercise, but only by ~ 1°C over 20 min (the approximate time of exercise in our study), regardless of exercise intensity (Kunstetter et al., 2014). There is one study on impact of body temperature on brain stiffness in mice, which found softening in normothermia (36–38°C) compared to hypothermia (28–30°C), though this would be only a very small effect for a 1°C change in the range of normothermia (Bertalan et al., 2019). From this we conclude it is unlikely temperature played a significant role in our results, though possibly has some influence.

Interestingly, we show regionally-dependent changes to mechanical properties with exercise. The hippocampus, ACC, and dlPFC change significantly in stiffness, but not the thalamus. Additionally, only the dlPFC significantly changes in damping ratio. Previous studies have shown regional dependency of CBF immediately after exercise, reporting CBF changes to gray matter, hippocampus, and insula, but not to regions including the caudate or the pre- or post-central gyrus (MacIntosh et al., 2014). We expect that the brain mechanical properties change regionally with exercise because of intrinsic differences in tissue microstructure and vasculature, which make them more or less mechanically responsive to blood flow stimuli. The degree of inter-subject variability in regional response to exercise is supported by the hypothesis that individuals have different mechanical responses to blood flow, and ultimately may explain the correlations we see between brain mechanical properties and improvements to reaction time.

We also find that the average reaction time on the Flanker and Stroop tasks are significantly improved with exercise suggesting an improvement in processing speed following an acute bout of exercise. Between TP1 and TP2, the exercise group improved 11.6% in response time to Flanker congruent stimuli, while the control group only improved 4.6%, likewise the exercise group improved 15.3% in response time on Stroop congruent stimuli, compared to the control group of just 2.4%. These findings are consistent with previous studies investigating the influence of exercise on processing speed (McMorris & Hale, 2012; Tomporowski, 2003). These data are also consistent with previous studies that have reported notable correlations between acute exercise and improved cognitive function (Audiffren et al., 2008; Brisswalter et al., 2002; Joyce et al., 2009; Tomporowski & Ellis, 1986; Tomporowski et al., 2008) and changes in reaction time during cognitive assessments (Hogervorst et al., 1996; Mcmorris & Peter Keen, 1994; Travlos & Marisi, 1995). Interestingly, we also demonstrate that for some brain regions, magnitude of change in stiffness is related to magnitude of improvement in reaction time. Previous MRE studies have shown notable correlations between cognitive function and regional tissue mechanical properties (Hiscox et al., 2020; Johnson et al., 2018; Schwarb et al., 2019), and that brain mechanical properties are potential mediators in the relationship between aerobic fitness and cognitive function (Schwarb et al., 2017). The ability to acutely alter brain mechanical properties marks a critical step towards designing and implementing neuromechanical rehabilitation strategies, particularly if these acute changes to mechanical properties reflect acute improvements to cognitive function.

There were several limitations to this study. Due to the limited time window for imaging after exercise, we were unable to collect ASL measures of CBF, making it impossible to probe the link between blood flow and brain mechanics. Previous studies have shown that baseline fitness levels can moderate effects of acute exercise, but we did not record measures of baseline fitness in our study (Schulz et al., 2004; Tomporowski & Ellis, 1986; White & Castellano, 2008; Zoladz et al., 2008). Previous studies have also shown that intensity of exercise relates to cerebral blood flow and cognitive performance post-exercise (Chang et al., 2012). Our exercise regimen was designed to be of high intensity and in the future, it would be advantageous to additionally assess brain mechanical properties at lower exercise intensities to determine if the responses are modulated. We did not find significant correlations between changes in brain stiffness and heart rate during exercise, though cardiovascular effects including heart rate and blood pressure could have an indirect influence on the results. The potential associations between brain stiffness and these variables in acute exercise should be examined and considered in future studies. Other factors known to influence performance on cognitive tasks, including education and socioeconomic status, were not measured in this study or considered in the analyses of how cognitive performance changes with acute exercise. When examining associations between changes in brain mechanical properties and improvement in reaction time following acute exercise, we found significant correlations in several regions expected to be important for task performance, however we did not explicitly correct for multiple comparisons. Finally, this study was restricted to young adults to allow us to study the link between cognitive function and brain mechanics prior to any neurodegeneration. In the future we hope to replicate these experiments in older adults with and without cognitive impairments.

## Conclusion

MRE offers a noninvasive imaging method through which to measure acute changes to underlying tissue mechanics, such as from acute exercise. Here we find significant decreases to brain stiffness and significant increases to brain damping ratio, which we believe result from a mechanical cascade starting with altered cerebral blood flow, which alters tissue microstructure in the short-term. We find these effects occur on a regional basis, which may be a result of the varying degrees of tissue vasculature in different brain regions and the inherent differences in microstructural response to mechanical stimuli. We find that with exercise, subjects improve in reaction time on the Eriksen Flanker and Stoop tasks, and the magnitude of that change is related to the magnitude of mechanical property change. Understanding the relationship between acute exercise and increased cognitive performance is valuable towards utilizing exercise training as a tool for improvement of brain structure and health.

## Supplementary Material

supplement

## Data Availability

Numerical data will be made available by simple request. Raw image files will be made available by request which includes a formal project outline and an agreement of data sharing.
