# Supplementary material for: Acute effects of high-intensity exercise on brain mechanical properties and cognitive function
Source: Brain Imaging Behav. Author manuscript; Available in PMC 2024 Sep 1. (PMC11364612; doi:10.1007/s11682-024-00873-y)
Supplement: supplement [file NIHMS1988650-supplement-supplement.zip › Acute Exercise & Brain Mechanical Properties Tasks/Stroop_Practice.html]

PsyToolkit run experiment
