# Supplementary material for: Acute effects of high-intensity exercise on brain mechanical properties and cognitive function
Source: Brain Imaging Behav. Author manuscript; Available in PMC 2024 Sep 1. (PMC11364612; doi:10.1007/s11682-024-00873-y)
Supplement: supplement [file NIHMS1988650-supplement-supplement.zip › Acute Exercise & Brain Mechanical Properties Tasks/README.rtf]

Acute Effects of High-Intensity Exercise on Brain Mechanical Properties and Cognitive FunctionG McIlvain, EM Magoon, RG Clements, A Merritt, LV Hiscox, H Schwarb, CL JohnsonNotes for Administering the Stroop TaskPrior to starting the task, keyboard should be marked with colored indicators on the appropriate keys. For the attached file the following characters on a US keyboard correspond to the following colors:Q = RedE = GreenI = YellowP = BlueStroop_Practice.html contains 15 trials. Stroop_Full.html contains 100 trials. Results are reported via the “Show Data” option at the end of the task as follows:Column 1 = N/AColumn 2 = Stimulus TextColumn 3 = Stimulus Text ColorColumn 4 = Congruency (0 = Incongruent, 1 = Congruent)Column 5 - 7 = N/AColumn 8 = Response Time (ms)Stroop Effect = Incongruent Response Time - Congruent Response TimeNotes for Administering the Flanker TaskThe X and V keys correspond to themselves but we included sticker indicators on these two keys to help participants ignore all other keys.Flanker_Practice.html contains 15 trials. Flanker_Full.html contains 100 trials. Results are reported via the “Show Data” option at the end of the task as follows:Column 1 = StimulusColumn 2 = Congruency (0 = Incongruent, 1 = Congruent)Column 3 = N/AColumn 4 = Response Time (ms)Flanker Effect = Incongruent Response Time - Congruent Response Time
